# MERS Coronavirus Neutralizing Antibodies in Camels, Eastern Africa, 1983–1997

**DOI:** 10.3201/eid2012.141026

**Published:** 2014-12

**Authors:** Marcel A. Müller, Victor Max Corman, Joerg Jores, Benjamin Meyer, Mario Younan, Anne Liljander, Berend-Jan Bosch, Erik Lattwein, Mosaad Hilali, Bakri E. Musa, Set Bornstein, Christian Drosten

**Affiliations:** University of Bonn Medical Centre, Bonn, Germany (M.A. Müller, V.M. Corman, B. Meyer, C. Drosten);; German Centre for Infection Research, Bonn (V.M. Corman);; International Livestock Research Institute, Nairobi, Kenya (J. Jores, A. Liljander);; Vétérinaires Sans Frontières Germany, Nairobi (M. Younan);; Utrecht University, Utrecht, the Netherlands (B.-J. Bosch); EUROIMMUN AG, Lübeck, Germany (E. Lattwein);; Cairo University, Giza, Egypt (M. Hilali);; Ministry of Science and Communication, Khartoum, Sudan (B.E. Musa);; National Veterinary Institute, Uppsala, Sweden (S. Bornstein)

**Keywords:** dromedary camel, reservoir, Middle East respiratory syndrome MERS, coronavirus, antibody, viruses, Africa

## Abstract

To analyze the distribution of Middle East respiratory syndrome coronavirus (MERS-CoV)–seropositive dromedary camels in eastern Africa, we tested 189 archived serum samples accumulated during the past 30 years. We identified MERS-CoV neutralizing antibodies in 81.0% of samples from the main camel-exporting countries, Sudan and Somalia, suggesting long-term virus circulation in these animals.

Since 2012, a newly emerged human pathogenic coronavirus (CoV) has caused an ongoing epidemic on the Arabian Peninsula. The designated Middle East respiratory syndrome (MERS)-CoV belongs to the *Betacoronavirus* genus lineage C and causes severe respiratory disease in humans (*1*). As of July 2, 2014, MERS-CoV has caused ≈842 human infections, including 322 deaths (*2*). Dromedary camels are a putative source for MERS-CoV infection in humans. Dromedaries from countries in Africa (Egypt, Tunisia, Nigeria, Sudan, Ethiopia, and Kenya) and Arabia (United Arab Emirates, Saudi Arabia, Oman, Qatar, and Jordan) have shown high rates of MERS-CoV seropositivity in serum samples collected during the past 2 decades (*3*–*9*). In addition, MERS-CoV nucleotide sequences and virus were detected in respiratory swab samples, predominantly from juvenile dromedaries (*5*,*10*). Transmission between humans and camels has been described in Qatar and Saudi Arabia (*11*,*12*). No autochthonous MERS-CoV infections in humans have been reported in Africa. Most dromedary camels traded in the Middle East are bred in the Greater Horn of Africa, primarily in Ethiopia, Sudan, Somalia, and Kenya (*13*). To further analyze the spatial and temporal distribution of MERS-CoV–seropositive camels, we tested archived camel serum samples originating in Egypt, Sudan, and Somalia, accumulated during the past 30 years, for MERS-CoV antibodies.

## The Study

A serum sample from each of 189 dromedary camels was collected by trained personnel as previously described (*14*). Blood samples were taken by jugular vein puncture. The blood was allowed to clot and subsequently centrifuged to obtain serum, or serum was separated from the coagulated blood during slaughter. All serum samples were heat-inactivated at 56°C for 30 min (*14*). Serum from Somalia was collected during 1983 and 1984; samples from Sudan were collected during June and July 1984, and samples from Egypt were collected during June and July 1997. All camels from Sudan were female (>6 years of age) and belonged to the Anafi breed. They were kept locally and used as a means of transport and a source of milk. The camels from Somalia were sampled at slaughterhouses in Afgoi and Mogadishu. Most camels were adults; however, detailed information about sex and age was not available. The camels from Somalia were bred predominantly for milk and meat. No background information was available for the camels from Egypt. Our study fully complied with national regulations and was approved by the ethics committee of the International Livestock Research Institute accredited by the National Council of Science and Technology in Kenya (approval no. ILRI-IREC2013–12).

We tested all serum samples for MERS-CoV antibodies at a 1:100 dilution by a recombinant MERS-CoV spike protein subunit 1–based ELISA (rELISA) as previously described (*3*,*12*). To determine the assay-specific cutoff value, we tested 124 confirmed MERS-CoV antibody–negative and 106 MERS-COV antibody–positive camel serum samples from previous studies (*3*). For inter-assay calibration, we used the same selected positive serum samples in all applications. The optical density (OD) was measured at 450/605 nm. We determined the OD ratio by dividing the OD of each sample by the OD of the positive serum. The cutoff was defined as the 3-fold mean OD ratio of all tested MERS-CoV antibody–negative serum samples (Technical Appendix Figure 1). To confirm antibody specificity and rule out possible cross-reactivity with other livestock-associated CoVs, we conducted a highly specific MERS-CoV microneutralization test (*3*,*6*). All serum samples were tested at a 1:80 dilution and at a 1:800 dilution to identify MERS-CoV neutralizing antibodies. Serum without neutralizing activity at 1:80 was rated MERS-CoV antibody negative.

A total of 159 (84.1%; range among countries 80.0%–86.7%) of 189 dromedary camels were positive for MERS-CoV antibodies in the rELISA (Figure, Table 1). The highly specific neutralization test confirmed that 153 (81.0%; range 68.0%–86.9%) of camels had neutralizing activity with reciprocal titers of >80 (Table 1). Whereas most samples (124 [65.6%; range 56.0%–73.8%]) had reciprocal neutralizing titers of 80–800, we detected high neutralizing titers of >800 in 29 (15.3%; range 11.6%–21.7%) samples (Table 2). Neutralizing titers correlated significantly (p<0.001, Kruskal-Wallis 1-way analysis of variance) with the determined OD ratios of the rELISA. The rELISA was 99.0% specific when correlated with the results of the microneutralization test (Technical Appendix Figure 2).

**Figure:**
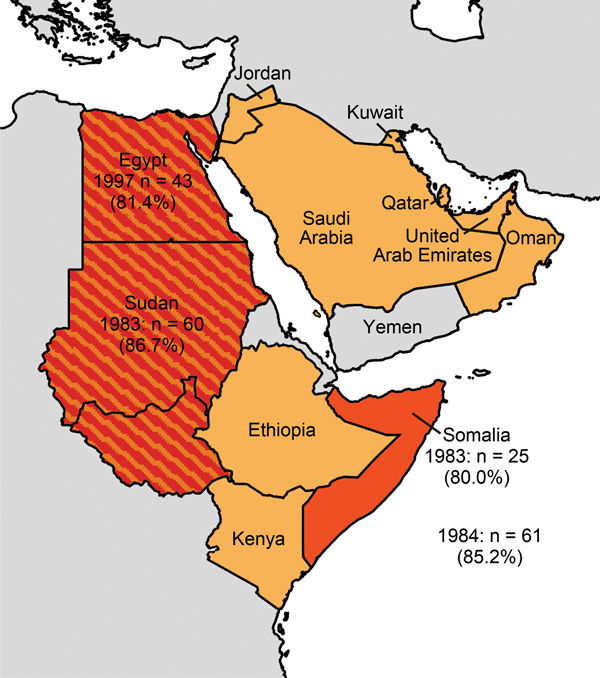
Arabian Peninsula and neighboring countries of the Greater Horn of Africa in 2014. The study sites Egypt, Sudan (separated into Sudan and South Sudan), and Somalia are in dark orange and labeled with the year the camels were sampled, the number of samples, and the percentage of samples that were reactive in the MERS-CoV ELISA. Countries with previously reported MERS-CoV seropositive dromedaries are in light orange (overlap shown in stripes).

**Table 1:** MERS-CoV antibodies in dromedary camels from eastern Africa*

Country, date	No. samples	No. rELISA positive (%)†	No. mNT positive (%)‡
Egypt, 1997 Jul	43	35 (81.4)	34 (79.1)
Somalia			
1983 Jan–Nov	25	20 (80.0)	17 (68.0)
1984 Feb–Dec	61	52 (85.2)	53 (86.9)
Sudan, 1983 Jun	60	52 (86.7)	49 (81.7)
Total	189	159 (84.1)	153 (81.0)

*MERS-CoV, Middle East respiratory syndrome coronavirus; mNT, microneutralization test; rELISA, recombinant ELISA based on the MERS-CoV subunit 1 spike protein. †Serum was tested at a dilution of 1:100. ‡mNT for MERS-CoV was done in a microtiter plate format in duplicate at dilutions 1:80 and 1:800. Serum with reciprocal titers >80 were considered MERS-CoV antibody positive.

**Table 2:** MERS-CoV neutralizing antibody titers in dromedary camels from eastern Africa*

Country, date	No. samples	mNT titer, no. (%) samples†		
		<80	80–800	>800
Egypt, 1997 Jul	43	9 (20.9)	29 (67.4)	5 (11.6)
Somalia				
1983 Jan–Nov	25	8 (32.0)	14 (56.0)	3 (12.0)
1984 Feb–Dec	61	8 (13.1)	45 (73.8)	8 (13.1)
Sudan, 1983 Jun	60	11 (18.3)	36 (60.0)	13 (21.7)
Total	189	36 (19.0)	124 (65.6)	29 (15.3)

*MERS-CoV, Middle East respiratory syndrome coronavirus; mNT, microneutralization test. †mNT for MERS-CoV was done in a microtiter plate format in duplicate at dilutions 1:80 and 1:800. Serum with reciprocal titers >80 were considered MERS-CoV antibody positive.

MERS-CoV antibody–carrying dromedaries were present in all 3 countries in 1983, 1984, and 1997 (Figure; Tables 1, 2). The high seropositivity in camels from Egypt (35 [81.4%] of 43), a country that imports camels from Sudan and Somalia, was consistent with previous studies (*7*,*10*). Strikingly, camels sampled in Somalia and Sudan >30 years ago were identified as MERS-CoV antibody positive with seropositivity of up to 86.7% in Sudan in 1983.

## Conclusions

Our study complements and supports the latest findings on long-term and widespread circulation of MERS-CoV or MERS-like CoV in dromedaries in Africa (*3*,*7*,*9*,*10*). By identifying neutralizing antibodies for MERS-CoV in Somalia dromedaries, we provided data for the country lodging the world's largest camel population and from which many camels are exported to Saudi Arabia (*13*). The large proportion of adult animals tested in this study explains the high seropositivity (>80%) and agrees with previous observations (*3*,*6*,*12*). Earlier reports provided evidence for seropositive camels in Kenya and Saudi Arabia dating to the early 1990s (*3*,*4*). Here we describe the presence of anti-MERS-CoV antibodies in archived serum collected >30 years ago, increasing the timescale for detection by an additional decade. Long-term circulation of MERS-CoV or MERS-like CoV in dromedaries can therefore be hypothesized. As suggested, an important factor possibly contributing to continuous virus maintenance in camels could be a high camel population density combined with nomadic husbandry, including frequent contact among camel herds in the Greater Horn of Africa (*3*).

MERS-CoV sequences from camels in Saudi Arabia and Qatar were closely related to sequences found in humans and did not show major genetic variability that would support long-term evolution of MERS-CoV in camels (*10*,*11*). The MERS-CoV sequence from a camel in Egypt was phylogenetically most distantly related to all other known camel-associated MERS-CoVs but closely related to the early human MERS-CoV isolates (*10*). An urgent task would be to characterize the diversity of MERS-related CoV in other camels in Africa to elucidate whether the current epidemic MERS-CoV strains have evolved toward more efficient human transmissibility.

The existence of unrecognized human infections in African or Arabian countries in the past cannot be ruled out. Resource-limited African countries that have been exposed to civil unrest, such as Somalia and Sudan, are not likely to diagnose and report diagnostically challenging infections resembling other diseases. The lack of MERS-CoV antibodies in a small cohort serosurvey in Saudi Arabia did not suggest the long-term circulation of MERS-CoV in humans on the Arabian Peninsula (*15*). Large serosurveys in countries where camels are bred and traded, especially in eastern Africa, are needed to explore the general MERS-CoV seroprevalence in camels and humans, particularly humans who have close contact with camels. Such serosurveys could provide the data needed to ascertain whether MERS-CoV has been introduced into, but unrecognized in, the human population on the African continent.

Technical Appendix Determination of the Middle East respiratory syndrome coronavirus (MERS-CoV) recombinant ELISA (rELISA) cutoff and correlation of the MERS-CoV rELISA and the virus neutralization test.
