# Supplementary material for: MERS Coronavirus Neutralizing Antibodies in Camels, Eastern Africa, 1983–1997
Source: Emerg Infect Dis. 2014 Dec;20(12):2093–5. doi: 10.3201/eid2012.141026 (PMC4257824; doi:10.3201/eid2012.141026)
Supplement: Technical Appendix — Determination of the Middle East respiratory syndrome coronavirus (MERS-CoV) recombinant ELISA (rELISA) cutoff and correlation of the MERS-CoV rELISA and the virus neutralization test. [file 14-1026-Techapp-s1.pdf]

Article DOI: http://dx.doi.org/10.3201/eid2012.141026

MERS Coronavirus Neutralizing Antibodies in Camels, Eastern Africa, 1983–1997

Technical Appendix

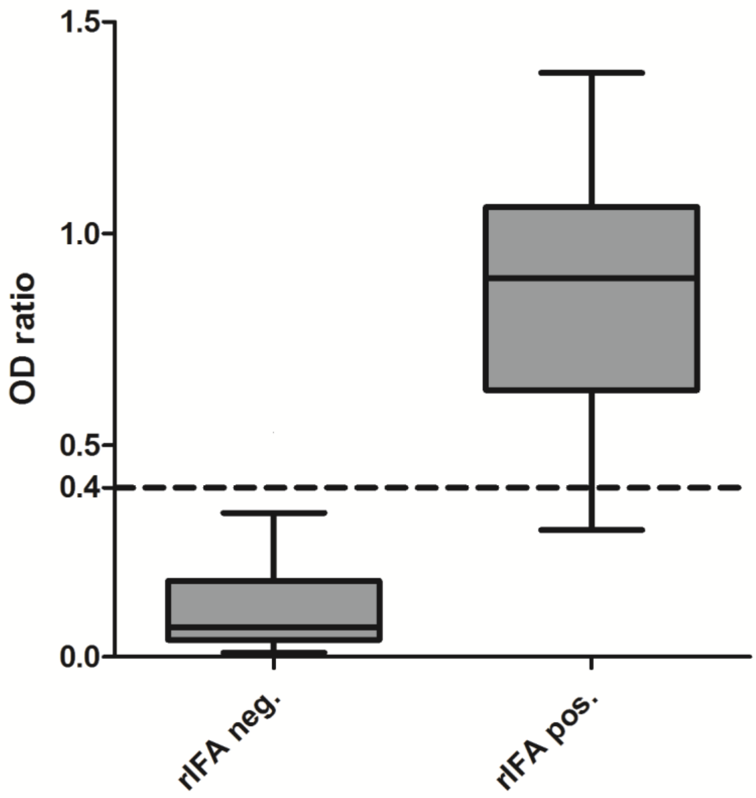

Technical Appendix Figure 1. Determination of the MERS-CoV rELISA cutoff. For determining the assay-specific cutoff, 124 dromedary camel serum samples that were previously confirmed to be MERS-CoV antibody negative (tested by rIFA) and 106 MERS-CoV rIFA-confirmed positive serum samples were tested in the MERS-CoV rELISA. The cutoff was defined as the 3-fold mean OD ratio of the negative serum measured at 450/605 nm (dashed line, OD ratio = 0.4). Data are shown as a box-and-whisker diagram. Boxes represent 50% of the complete data set comprising the first and third quartiles. Upper and lower whiskers indicate the highest and lowest value excluding outliers (Tukey method). The horizontal black line represents the median. MERS-CoV, Middle East respiratory syndrome coronavirus; rELISA, recombinant ELISA; rIFA, recombinant immunofluorescence assay; OD, optical density; neg, negative; pos, positive.

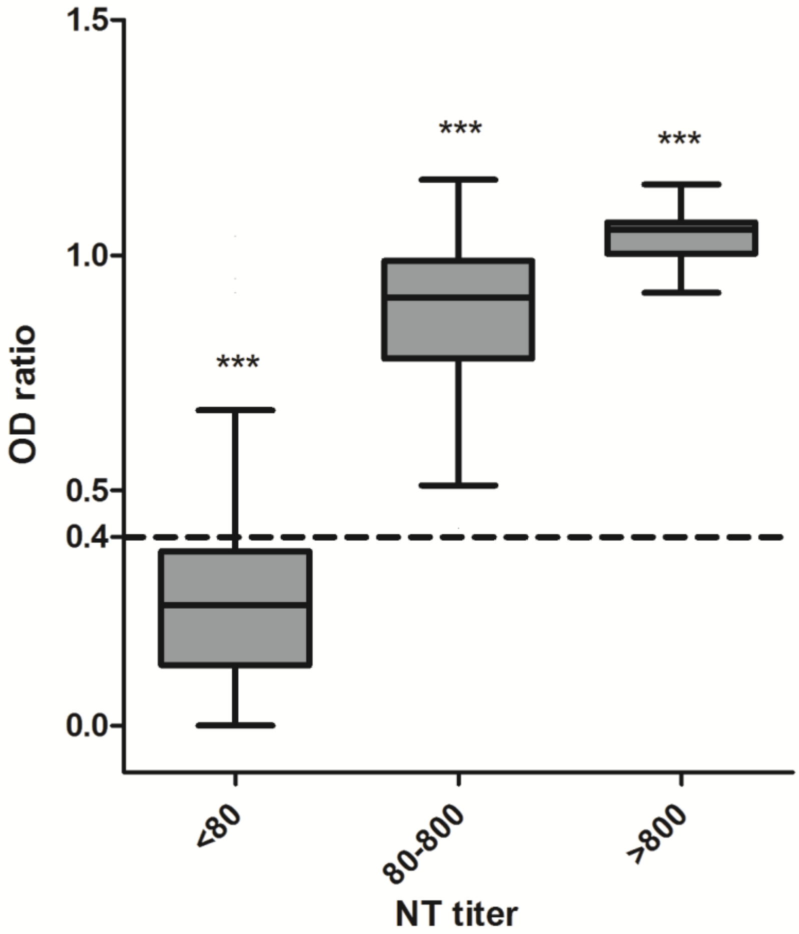

Technical Appendix Figure 2. Correlation of the MERS-CoV rELISA and the virus NT. Neutralizing antibody titers of camel serum were plotted against the OD ratios measured at 450/605 nm in the MERS-CoV rELISA. The OD ratio = 0.4 was used as the assay-specific cutoff and is shown as a dashed line. Data are shown as a box-and-whisker diagram. Whiskers of the box plot indicate the maximum and minimum values excluding outliers (Tukey method). The median is represented by a horizontal black line. Statistical analysis was done by Kruskal-Wallis 1-way analysis of variance. ***p<0.001. MERS-CoV, Middle East respiratory syndrome coronavirus; rELISA, recombinant ELISA; NT, neutralization test; OD, optical density.
